# Early-Life Gut Microbiota: Education of the Immune System and Links to Autoimmune Diseases

**DOI:** 10.3390/microorganisms14010210

**Published:** 2026-01-16

**Authors:** Pleun de Groen, Samantha C. Gouw, Nordin M. J. Hanssen, Max Nieuwdorp, Elena Rampanelli

**Affiliations:** 1 Department of (Experimental) Vascular Medicine, Amsterdam UMC Location University of Amsterdam, 1105 AZ Amsterdam, The Netherlands; p.degroen@amsterdamumc.nl (P.d.G.); n.m.j.hanssen@amsterdamumc.nl (N.M.J.H.); m.nieuwdorp@amsterdamumc.nl (M.N.); 2 Diabeter Center Amsterdam, 1066 EC Amsterdam, The Netherlands; 3 Department of Pediatric Hematology, Amsterdam UMC Location University of Amsterdam, 1105 AZ Amsterdam, The Netherlands; s.c.gouw@amsterdamumc.nl; 4 Amsterdam Institute for Infection and Immunity (AII), 1105 AZ Amsterdam, The Netherlands

**Keywords:** gut microbiota, early-life microbiome, immune development, dysbiosis, long-term health and disease

## Abstract

Early life is a critical window for immune system development, during which the gut microbiome shapes innate immunity, antigen presentation, and adaptive immune maturation. Disruptions in microbial colonization—driven by factors such as cesarean delivery, antibiotic exposure, and formula feeding—deplete beneficial early-life taxa (e.g., *Bifidobacterium*, *Bacteroides*, and *Enterococcus*) and impair key microbial functions, including short-chain fatty acid (SCFA) production by these keystone species, alongside regulatory T cell induction. These dysbiosis patterns are associated with an increased risk of pediatric autoimmune diseases, notably type 1 diabetes, inflammatory bowel disease, celiac disease, and juvenile idiopathic arthritis. This review synthesizes current evidence on how the early-life microbiota influences immune maturation, with potential effects on the development of autoimmune diseases later in life. We specifically focus on human observational and intervention studies, where treatments with probiotics, synbiotics, vaginal microbial transfer, or maternal fecal microbiota transplantations have been shown to partially restore a disrupted microbiome. While restoration of the gut microbiome composition and function is the main reported outcome of these studies, to date, no reports have disclosed direct prevention of autoimmune disease development by targeting the early-life gut microbiome. In this regard, a better understanding of the early-life microbiome–immune axis is essential for developing targeted preventive strategies. Future research must prioritize longitudinal evaluation of autoimmune outcomes after microbiome modulation to reduce the burden of chronic immune-mediated diseases.

## 1. Introduction

The immune system is fundamental to human health, orchestrating defense against pathogens while maintaining tolerance to self-tissues. In autoimmune diseases, this delicate balance is disrupted, leading to inappropriate immune responses against the body’s own cells. Recent research has indicated that the gut microbiome, defined as the vast and diverse community of microorganisms inhabiting the gastrointestinal tract, is a central player in immune system development and regulation [1,2]. Far from passive symbionts, gut microbes educate both innate and adaptive immunity via metabolites (SCFAs, indole-3-lactic acid) and signaling, promoting immune cell differentiation, regulatory T cell induction, and immunoglobulin A (IgA) production [1,3].

**Figure 1:**
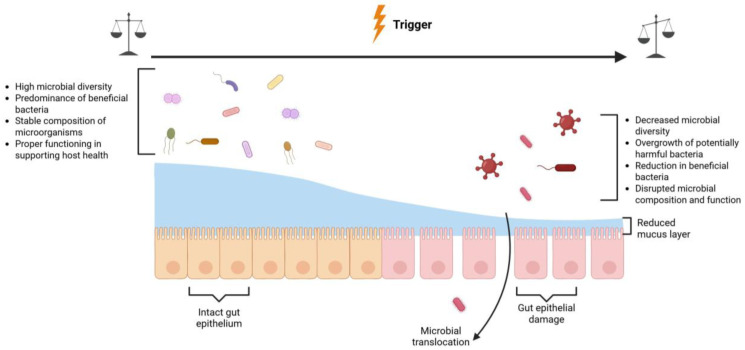
Diagram illustrating the effects of a stable/diverse and an unstable/disrupted microbiome composition. High microbial species diversity and diverse metabolic functions, as well as the predominance of beneficial bacteria (e.g., *Bifidobacterium* and *Lactobacillus*), support a symbiotic relationship between gut microbes and their host and promote host health. Triggers such as antibiotics, infections, or dietary changes can disrupt this balance, resulting in reduced microbial diversity, increased abundance of potentially harmful taxa, loss of beneficial bacteria (e.g., *Bifidobacterium*, *Bacteroides*), and diminished gut barrier integrity. Image created with BioRender.com (accessed on 12 January 2026).

Alterations in the composition or function of the gut microbiota, particularly during the critical early-life window, have been linked to immune dysregulation and increased risk for a range of chronic conditions, including autoimmune diseases, allergies, and metabolic disorders [4]. Mechanistically, the microbiome interacts with the immune system through direct microbial signaling, production of metabolites, and modulation of the gut barrier, thereby influencing inflammatory and regulatory pathways. A balanced and diverse gut microbiota supports immune health by maintaining microbial stability and beneficial bacteria, whereas disruptions—such as those triggered by antibiotics, infections, or dietary changes—can result in reduced diversity, dominance of potentially harmful bacteria, and impaired epithelial integrity, often referred to as dysbiosis (see Figure 1).

This literature review explores the role of the early-life gut microbiome in shaping immune system development and its potential influence on the risk of autoimmune diseases. We hypothesize that the formation of a diverse and balanced gut microbiome from the prenatal period through early childhood is essential for optimal immune function and that disruptions to this process increase the likelihood of autoimmune pathology later in life. By synthesizing current evidence, this review aims to clarify the mechanisms underlying microbiome–immune interactions and identify opportunities for early interventions to promote lifelong health.

## 2. Materials and Methods

This comprehensive review synthesizes data from preclinical and clinical studies exploring the influence of the early-life gut microbiome on immune system development and autoimmune disease risk. Relevant literature was identified exclusively through systematic searches of the PubMed database up to October 2025. Search terms included “gut microbiome,” “early-life,” “immune system development,” “immune tolerance,” “autoimmune diseases“ and “microbial metabolites.”

Only findings from peer-reviewed articles published in English were included in this review. Both original research articles and reviews were included to provide a thorough overview of current understanding and emerging insights. To capture the translational and clinically relevant perspectives, we prioritized the description of findings from human cohorts or human clinical trials. When applicable, references to public datasets and clinical trial registries from individual studies were noted.

Generative artificial intelligence (GenAI) tools were used solely for language editing and formatting assistance, without contributing to the scientific content, data analysis, or interpretation of this manuscript.

## 3. The Development of the Early-Life Microbiome (0–3 Years)

The establishment of the gut microbiome during infancy is a highly dynamic and critical process, with profound implications for lifelong health. Compared to adults, the infant gut microbiota is characterized by markedly lower diversity—up to six times fewer operational taxonomic units (OTUs)—yet it undergoes rapid compositional changes throughout the first three years of life [5,6,7,8,9]. The predominant bacterial phyla in the infant gut include Actinobacteria (mainly *Bifidobacterium*), Proteobacteria (primarily unclassified *Enterobacteriaceae*), Firmicutes (such as *Streptococcus* and *Enterococcus*), and Bacteroidetes (notably *Bacteroides*) [10].

Traditionally, it was believed that microbial colonization of the infant gut began at birth. However, recent studies have detected the presence of microbial DNA in the placenta, amniotic fluid, umbilical cord, and meconium, thereby challenging the prevailing concept of a sterile in utero environment [11,12,13]. Despite these findings, the existence and significance of prenatal microbial presence remain subjects of ongoing debate, due to the challenges in identifying low-biomass microbial communities and the lack of established methodologies to detect false positivity due to contamination of low-bacterial-abundance samples during processing [14,15,16].

**Figure 2:**
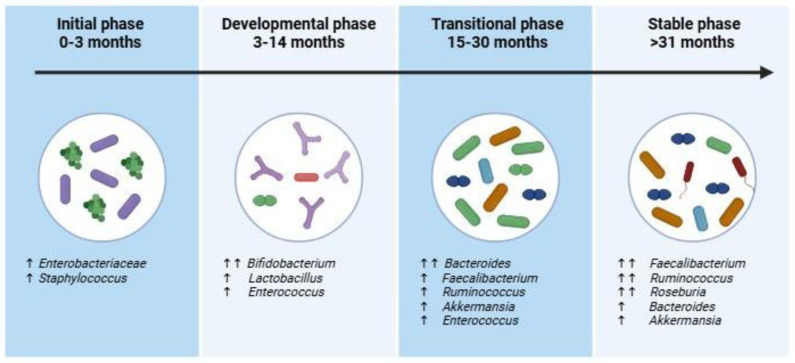
Developmental succession of the gut microbiome during early life. The figure illustrates dynamic changes in dominant bacterial taxa in stool samples, which represents the colonic microbiota, across four phases: the initial phase (0–3 months) marked by a prevalence of *Enterobacteriaceae* and *Staphylococcus*; the developmental phase (3–14 months) characterized by increased *Bifidobacterium*, *Lactobacillus*, and *Enterococcus*; the transitional phase (15–30 months) featuring a rise in *Bacteroides*, *Faecalibacterium*, *Ruminococcus*, *Akkermansia*, and *Enterococcus*; and the stable phase (>31 months), where *Faecalibacterium*, *Ruminococcus*, *Roseburia*, *Bacteroides*, and *Akkermansia* predominate. These sequential shifts reflect the maturation and stabilization of the gut microbial community during early childhood. Image created with BioRender.com (accessed on 12 January 2026).

Immediately after birth, the neonatal gut is initially colonized by facultative anaerobes, such as *Enterobacteriaceae* and Staphylococcus species, due to the presence of oxygen in the newborn intestine [6,17,18]. As the gut environment becomes increasingly anaerobic, obligatory anaerobes begin to dominate. Longitudinal studies, such as the TEDDY study, defined three distinct phases of fecal microbiome maturation based on fecal sample analysis: the developmental phase (3–14 months), the transitional phase (15–30 months), and the stable phase (from 31 months onwards) (see Figure 2) [7].

In the developmental phase, the gut microbiome is dominated by *Bifidobacterium* species, including *B. breve*, *B. bifidum*, and *B. longum*, which are particularly prevalent in breastfed infants [6,19,20]. These bacteria play a crucial role in gut and immune health through the production of short-chain fatty acids (SCFAs). Human milk oligosaccharides (HMOs) in breast milk function as selective prebiotics, fostering the growth of *Bifidobacterium* and shaping the early microbial landscape [21,22]. Other genera, such as *Lactobacillus* and *Enterococcus*, are present in lower abundance, while *Escherichia coli* and *Streptococcus* species are also detected but remain less dominant [23].

The transitional phase is characterized by a significant increase in microbial diversity, which occurs concurrently with the introduction of solid foods and the emergence of a more extensive array of bacterial taxa [24]. This phase is characterized by a shift towards a more adult-like gut microbiota composition, marked by the prominence of *Bacteroides* species, particularly *B. fragilis*, *B. vulgatus*, and *B. thetaiotaomicron* [7,25,26,27]. The diversification of the microbiome during this phase is essential for the establishment of a balanced and resilient gut ecosystem.

By the stable phase, the gut microbiome reaches a mature and relatively stable state, characterized by increased alpha diversity and consistent microbial communities [7,24,28]. Firmicutes become the dominant phylum, with key genera such as *Faecalibacterium*, *Roseburia*, and *Ruminococcus* contributing to fiber fermentation and SCFA production processes vital for maintaining gut health and immune function. Genera such as *Bacteroides* and *Akkermansia* also remain integral to the microbiome’s complexity and stability.

## 4. Factors That May Influence the Composition of the Microbiome in the Early Stages of Life

### 4.1. Maternal Factors

Maternal nutrition, antibiotic use, gestational age, and mode of delivery correlate with distinct differences in neonatal microbiome composition.

Mode of delivery shows strong associations with neonatal microbial inheritance: vaginal delivery correlates with maternal vaginal/gut taxa, including *Lactobacillus*, *Prevotella*, and *Bacteroides*, whereas cesarean section (CS) correlates with skin/environmental microbes, such as *Staphylococcus*, *Corynebacterium*, and *Propionibacterium*, alongside delayed *Bacteroides* and *Bifidobacterium* colonization [29,30]. Dominguez-Bello et al.’s prospective cohort (*n* = 46 newborns) observed vaginal-birth microbiota resembling maternal vaginal profiles (*Lactobacillus*/*Prevotella*-dominant), while CS infants exhibited skin-like *Staphylococcus*/*Corynebacterium* enrichment across body sites [29]. The HELMi cohort (*n* = 984 Finnish infants) reported lower *Bifidobacterium*/*Bacteroides* abundance and α-diversity in CS versus vaginal births [31].

Intrapartum antibiotic prophylaxis (IAP)—routine in CS (postoperative infection prevention) and ~25% of vaginal deliveries (Group B Streptococcus prevention)—correlates with sustained microbiota alterations [32]. CHILD Study subsets (*n* = 198) linked IAP to reduced *Bacteroidaceae* and elevated *Clostridiales* through 12 months, partially attenuated by breastfeeding [33]. Finnish HELMi/Jorvi cohorts (*n* = 144) associated IAP with depleted *Bifidobacterium*/*Bacteroides* and enriched *Bacilli*/*Proteobacteria* (cephalosporins > penicillin) persisting for 1 year, independent of delivery/feeding and comparable to CS patterns [34]. Additionally, in a prospective cohort from the New Hampshire Birth Cohort Study (NHBCS, *n* = 266), it was found that ≥2 antibiotic classes correlated with reduced α-diversity/distinct structure at 6 weeks (GUnifrac *p* = 0.02), penicillin with slower *Bacteroides* rise, cephalosporins with delayed *Bifidobacterium*, and multi-class with *Veillonella dispar* enrichment [35].

Another factor is maternal nutrition. The MAMI study (*n* = 86) identified pregnancy gut clusters: *Prevotella*-dominant (high carbs, low fiber/ω-3) versus *Ruminococcus*-enriched (high plant proteins/fiber/PUFAs/polyphenols), associating with neonatal microbiota, growth (BMI/weight-for-length z-scores at 1/18 months), and inflammatory patterns varying by delivery mode [36]. Lundgren et al. (*n* = 145) linked higher maternal fruits/vegetables to elevated infant *Streptococcus*/*Clostridium* and lower Enterobacteriaceae at 6 weeks. [37]. Similarly, Chu et al. (*n* = 81) associated fat-rich maternal diets with meconium *Enterococcus* and 6-week Bacteroides abundance [38].

### 4.2. Environmental Factors

Postnatal environmental exposures strongly correlate with the composition and maturation trajectory of the infant gut microbiome.

Breastfeeding consistently correlates with *Bifidobacterium*-dominant microbial profiles, a pattern largely mediated by human milk oligosaccharides (HMOs) that selectively support taxa such as *Bifidobacterium longum* subsp. *infantis*, *B. breve*, and *B. bifidum* [39,40,41]. In the Cambridge Baby Growth and Breastfeeding Study (CBGS-BF; *n* = 94 mother–infant pairs), longitudinal profiling revealed that exclusive breastfeeding (EBF) beyond 6 months correlated with significantly higher *B. bifidum* abundance (*p* = 0.0285) compared to EBF < 3 months. Extended EBF also correlated with lower bacterial α-diversity but enrichment of short-chain fatty acid (SCFA)–related pathways [42]. Similar trends were reported in the Copenhagen Infant Gut (CIG) cohort (*n* = 25 exclusively breastfed infants), where fecal aromatic lactic acid concentrations correlated positively with the abundance of HMO-degrading *Bifidobacterium* species possessing aromatic lactate dehydrogenase, including *B. longum*, *B. breve*, and *B. bifidum* [22]. Evidence from a mother–infant transmission study (*n* = 21 exclusively breastfed dyads) indicated bidirectional transfer of *B. breve* between breast milk and the infant gut, suggesting direct microbial exchange [43]. Meta-analyses integrating 1825 samples from 684 infants further indicate that exclusive breastfeeding correlates with reduced bacterial α-diversity but enrichment of SCFA-producing bacteria and decreased *Enterobacteriaceae* abundance relative to formula feeding [44]. Conversely, formula feeding tends to correlate with increased relative abundances of opportunistic taxa such as *Clostridium difficile* and *Escherichia coli*, as well as mucus-degrading species like *Akkermansia muciniphila* and *Ruminococcus gnavus*. These microbial shifts often coincide with altered markers of intestinal barrier integrity [45]. In the HELMi cohort (*n* = 984 Finnish infants), prolonged breastfeeding (≥6 months) among cesarean-delivered infants correlated with increased *Bifidobacterium*/*Bacteroides* ratios, partially normalizing delivery mode-related dysbiosis toward the composition observed in vaginally delivered, breastfed infants [31].

Early life antibiotic exposure correlates strongly with disrupted microbial assembly, typically characterized by *Bifidobacterium* depletion and *Enterobacteriaceae* expansion. In preterm NICU cohorts, broad-spectrum regimens such as amoxicillin plus cefotaxime have been linked to sharp reductions in *Bifidobacterium* and *Lactobacillus*, accompanied by overgrowth of *Enterobacteriaceae* and other *Gammaproteobacteria*, with perturbations persisting for several weeks after treatment cessation [46]. A U.S. multi-NICU cohort (*n* = 236 very preterm infants) correlated early *Gammaproteobacteria* dominance with delayed colonization by obligate anaerobes despite milk feeding [47]. Similarly, the French EPIFLORE cohort (*n* = 159 preterm infants) identified persistently lower Shannon diversity at 3.5 years relative to term controls [48]. In the REASON randomized trial (*n* = 40 preterm neonates), antibiotic exposure correlated with reduced *Veillonella* abundance and lower fecal GABA concentrations, while positive *Veillonella–GABA* correlations persisted post-treatment [49]. Observations from the ZEBRA randomized trial (*n* = 147 infants ≥36 weeks GA) support these patterns, with early-life exposure to amoxicillin and cefotaxime correlating with the largest shifts in gut microbial composition and antibiotic resistance gene patterns—including decreased *Bifidobacterium* and increased *Klebsiella/Enterococcus*—relative to controls and other regimens [50].

Broader environmental exposures also correlate with distinct microbial signatures and maturation patterns. Pet ownership correlates with increased gut microbial richness in early infancy. In the Canadian CHILD Cohort (*n* = 746), pre- and postnatal furry pet exposure (including dogs) was linked to higher fecal species richness (Chao1) at 3 months, *Oscillospira*/*Ruminococcus* enrichment across birth modes, and reduced *Proteobacteria* [51]

Household endotoxin exposure correlates with greater *Bifidobacterium* species diversity (including *B. adolescentis*) in early infancy. In a Swedish birth cohort (*n* = 47), higher house dust endotoxin levels were associated with colonization by 3–4 *Bifidobacterium* species at 1 week and 2 months, compared to fewer species in low-endotoxin homes [52]

Sibling presence correlates with accelerated gut microbiota maturation. In the Danish SKOT I cohort (*n* = 114), more older siblings correlated with higher bacterial α-diversity (*p* = 0.030), richness (*p* = 0.006), Firmicutes (*p* = 0.013), and Bacteroidetes diversity (*p* = 0.004) at 18 months [53]. In the Dutch KOALA Birth Cohort (*n* = 1032 at 1 month), infants with older siblings had slightly higher fecal *Bifidobacterium* counts (*p* trend = 0.07) vs. only children [54].

Farm exposure correlates with accelerated anaerobic gut microbiota development and reduced pathogen carriage. In the Swedish FARMFLORA cohort (*n* = 65), farm-reared infants showed a seven-fold higher anaerobe/facultative ratio at 1 week (*p* = 0.020), lower *E. coli* counts when colonized during the first 4 months (*p* < 0.05), less frequent *C. difficile* colonization at 12 months (*p* < 0.001), and higher *Bifidobacterium*/*Lactobacillus* counts at 6 months (*p* = 0.032/0.027) vs. rural controls—all adjusted for pets, sex, and breastfeeding.

## 5. Impact of the Gut Microbial Colonization on the Neonatal Immune Development

**Figure 3:**
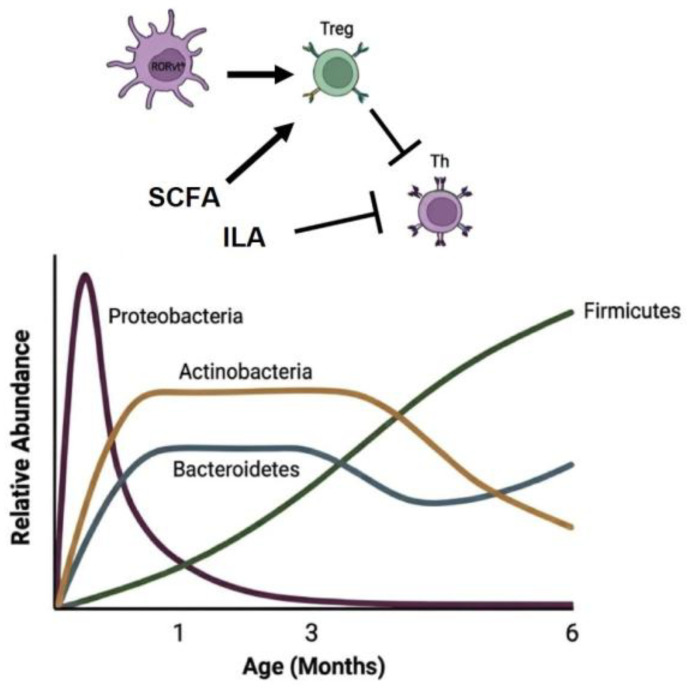
Development of gut microbiome and immune system during early life. The first colonizers are species of the family Enterobacteriaceae, which, by consuming oxygen, create an anaerobic environment. Within the first few weeks after birth, Bifidobacteria (obligate anaerobes of the Actinobacteria phylum) and species of Bacteroides dominate the gut microbiota as a consequence of breastfeeding. With the introduction of solid food between 4 and 6 months, there is a diversification of the gut microbiota composition, with an increasing abundance of diverse bacterial species, such as *Ruminococcus*, *Faecalibacterium prausnitzii*, *Bacteroides*, and *Prevotella* species. Through metabolite production in the first weeks after birth, Bifidobacteria “educate” the immune system to promote a tolerogenic intestinal milieu. The production of SCFAs (short-chain fatty acids) and ILA (indole lactic acid) by Bacteroides and Bifidobacteria species, in the first months of life, promotes the expansion of regulatory T cells (Treg) and anti-inflammatory effects on helper T cells (Th).

Early-life gut microbiota development occurs in parallel with the maturation of the immune system and is vital for training the developing immune system to establish tolerance and distinguish between commensal and pathogenic bacteria (Figure 3).

In this section, we therefore introduce the structure of the adult intestinal immune system and describe how early-life microbial colonization influences the development of the intestinal immune system.

The intestinal mucosal immune system is organized in three major compartments: the draining mesenteric lymph nodes (MLN), the gut-associated lymphoid tissues (GALT), and the effector sites within the intestinal lamina propria and intestinal epithelium layer. The GALT comprises Peyer’s patches (PPs) and isolated lymphoid follicles (ILF) and constitutes the site of lymphocyte priming and differentiation. After activation, effector T cells traffic to the intestinal epithelium and lamina propria, where they exert a pivotal role in maintaining immune homeostasis, tolerance to commensal bacteria and food antigens, and protection against enteropathogens [55].

PPs are localized in the small intestine, with the highest density in the ileum, and their development begins prenatally from around 14–16 weeks of gestation in humans. PPs are specialized sites for adaptive immune cell priming and differentiation; indeed, they contain B cell-rich follicles and perifollicular T cell zones enriched in central memory CD4+ T cells, FOXP3+ T regulator cells (Treg), and T follicular helper cells (Tfh) [56]. The sampling of luminal antigens is carried out by microfold M cells residing throughout the follicle-associated epithelium. M cells shuttle free and immunoglobulin A-bound antigens to antigen-presenting cells in the subepithelial compartment, allowing for lymphocyte priming in PP [57].

ILFs are smaller than PPs and are localized in both the small and large intestine [55]. In contrast to PP, ILF development begins in the first weeks after birth and is regulated by microbiota-derived clues that activate innate immune receptors, such as NOD1, and cytokine release [58]. The maturation of ILF in the small and large intestine seems to be differentially regulated. While small intestine ILF maturation depends on dietary and microbial products [58,59,60,61], large intestine ILF was shown to be promoted by IL-23 but inhibited by microbiota-induced IL-25 through inhibition of IL-23 [62].

While the development of the immune system begins in utero, the colonization of the body after birth is postulated to stimulate the maturation of the immune system. Nonetheless, evidence shows that maternal clues (including microbiota-derived signals) can influence the offspring’s early-life immunity. Notably, maternal immunoglobulins are transferred through the placenta and through breast milk to newborns and are pivotal for providing temporary immunity against pathogens [63]. In support of a role of maternal signals in shaping the intestinal immunity in offspring, a murine study disclosed that microbial colonization of pregnant dams induces an intestinal transcriptional reprogramming in the offspring, including enhanced expression of genes encoding antibacterial peptides, and shapes the neonatal intestinal immune landscape by increasing the proportions of group 3 innate lymphoid cells (ILC) and F4/80+ CD11c+ myeloid cells. Importantly, these changes were in part driven by the transfer of maternal immunoglobulins and microbial metabolites to the offspring and prevented inflammatory reactions against microbial products and penetration of gut microbes [64]. In line, another study in mice showed that a low-fiber diet during pregnancy altered the composition of the maternal milk microbiome and consequently of the neonatal gut microbiome. These microbial changes resulted in impaired production by epithelial cells of the Ftl3 ligand, a crucial growth factor in hematopoiesis, and consequently impaired hematopoiesis of plasmacytoid dendritic cell (pDC). Indeed, Ftl3 ligands drive the development of dendritic cells (DCs) and particularly of plasmacytoid dendritic cells (pDCs). Given that a low-fiber diet resulted in lower production of short-chain fatty acids, such as propionate, by gut microbes, the investigators supplemented dams with propionate or propionate-producing bacteria, which resulted in the restoration of both intestinal secretion of Ftl3 ligands and pDC hematopoiesis [65].

In support of a role of SCFA in the neonatal microbiome and particularly in the maturation of the immune system, feeding pregnant dams a standard-fiber-rich diet or a fiber-free diet impacted the microbiome assembly in the pups. The pups of fiber-deprived dams also exhibited alterations in the colonic immune landscape with increased expression of genes encoding IL-22 and involved in immune defense, along with a diminished proportion of RORγt+ (retinoic-acid-receptor-related orphan receptor-γt) innate and adaptive immune cells, which are required for intestinal immune homeostasis. These effects were attributed to the delayed colon colonization with *Akkermansia muciniphila* in pups of fiber-deprived dams [66]. Overall, these studies demonstrate the critical role of maternal microbiota and derived metabolites in shaping neonatal immune development.

The early postnatal period is considered an early “window of opportunity,” during which the neonatal immune system is shaped in response to microbial and dietary antigens. This period is characterized by the establishment of immunotolerance towards commensal microbes, setting the threshold for host-microbiome immune reactions in adult life [67]. Early activation and expansion of regulatory T cells prevent excessive immune responses to the environmental changes that newborns endure after birth. Hayakawa et al. reported that newborn babies exhibit an expansion of activated Treg in the early neonatal period (7–8 days after birth) as compared to the late neonatal period (2–4 weeks after birth). Interestingly, antenatal antibiotic exposure significantly decreased the proportion of activated Treg in the early neonatal phase, supporting the notion that early-life exposure to microbial antigens induces immunotolerogenic responses [68]. Moreover, several studies have demonstrated the ability of specific commensal bacteria to induce the differentiation and expansion of effector T cells. For instance, *Bacteroides* are pioneer commensals of the infant microbiome thanks to their ability to metabolize human milk oligosaccharides and have been shown to regulate T cell differentiation [69]. Monocolonization of germ-free mice with *Bacteroides fragilis* elicits immunotolerance by inducing the differentiation of regulatory FOXP3+ T cells in the gut and the production of the immunosuppressive cytokine IL-10 [70]. These effects could be attributed to the *B. fragilis* production of indole-3-lactic acid (ILA), which was found 10-fold more abundant in feces from *B. infantis* EVC001-treated children as compared to untreated children. ILA is a metabolite derived from microbial metabolism of tryptophan, which has been shown to exert an anti-inflammatory effect in the intestines [71,72,73].

The early-life expansion of Treg is regulated by specialized antigen-presenting cells (APC) expressing RORγt. RORγt+ innate lymphoid cells limit commensal bacteria-specific CD4 T cell responses through MHC-II-dependent interactions with CD4 T cells. Indeed, loss of RORγt+ ILC or of MHC-II expression on these cells dysregulates the immune responses to commensal bacteria, inducing intestinal inflammation in mice [74]. Specifically, MHCII+ ILC3s were shown to provoke cell death of commensal bacteria-specific activated T cells in mice, and expression of MHC-II on colonic ILC3 was found to be suppressed in children with inflammatory bowel disease, indicating a similar role of ILC3 in mice and humans [75]. Later studies have defined other subsets of RORγt+ APC, expressing AIRE or αvβ3 integrin (Janus and Thetis cells), which seed the intestinal lymph nodes in early life and are found sufficient and required to induce the differentiation of RORγt+Foxp3+ Treg and promote microbiota-specific T cell tolerance [76,77,78].

Alongside conventional effector T cells, unconventional T cells, such as invariant natural killer T cells (iNKT) and mucosal-associated invariant T cells (MAIT), seed the mucosal tissues in response to early-life colonization. This subset has a limited antigenic range due to the expression of semi-invariant T cell receptors. iNKT cells appear in the murine colon in the first 5–6 days after birth, and their colonic recruitment is dependent on resident fetal-derived macrophages [79]. Studies employing germ-free mice and bacteria monocolonization have revealed that the early-life microbiota establishment in the gut suppresses the expansion of colonic iNKT cells with persistent effects on NKT functions [80,81]. MAIT cells reside at the mucosal sites and are found in high frequency in the human lung and gut lamina propria. MAIT cells recognize vitamin B2 (riboflavin)-derived metabolites, which are also produced by some intestinal bacteria [82]. Their development in the thymus and expansion in extrathymic mucosal sites was shown to depend on microbiota-derived signals, given their relative deficiency in germ-free mice and the requirement of riboflavin-synthesizing commensal bacteria for the intrathymic development of MAIT cells [83,84,85]. Although the function of MAIT cells on systemic inflammatory responses is not fully elucidated, they contribute to barrier integrity and wound healing and are found to be decreased in the circulation in several autoimmune diseases [82,86].

Another mechanism governing the balance between effector proinflammatory and tolerogenic immune responses in the neonatal intestines is the spatial and temporal regulation of innate immune receptors (pattern-recognition receptors, PRR) that recognize microbiota-derived ligands. For instance, continuous signaling via toll-like receptor 4 (TLR4) in the murine intestinal epithelium induced by LPS persistence during the neonatal phase induces immune tolerance by triggering the transcriptional suppression of interleukin 1 receptor-associated kinase 1 (IRAK1) [87]. Instead, expression of TLR5, which recognizes flagellin, in neonatal intestines counteracts the colonization of flagellated bacteria by inducing the production of the antibacterial protein REG3γ and protects against the infection of flagellin-bearing pathogens [88,89].

Altogether, a growing body of literature links the early-life microbial colonization with immunomodulation and establishment of tolerance towards commensal microbes, imprinting the susceptibility to inflammatory reactions towards commensal microbes in adult life. Therefore, disruptions in the natural course of colonization after birth can negatively impact the intestinal and systemic immune landscape and influence the course of inflammatory diseases. In the next section, we will summarize the evidence from studies linking early-life perturbations in the gut microbiome to autoimmune diseases.

## 6. Early-Life Microbiome Alterations and Risk of Pediatric Autoimmune Disease

As outlined above, the early-life gut microbiome is essential for immune education and may therefore affect long-term immune homeostasis. Evidence about the importance of the crosstalk between gut microbes and the host derives from studies of diseased conditions versus the healthy state. There exists little consensus on what defines a healthy or diseased microbiome; nonetheless, key hallmarks of a disrupted microbiota have been described in multiple studies regardless, and they are associated with the onset of autoimmune diseases [90]. One key feature is the loss of keystone bacterial taxa, often following broad-spectrum antibiotic use. These keystone species are essential for maintaining microbial community structure and supporting immune maturation; their loss can lead to impaired immune regulation and increased vulnerability to infection and inflammation. A second hallmark is the reduction in microbial diversity, which has been linked to higher risks of infections, autoimmune diseases, and allergic disorders, consistent with the hygiene hypothesis. Third, the depletion of beneficial commensals creates ecological niches that may be filled by opportunistic pathogens or pathobionts, predisposing individuals to both acute and chronic conditions. Finally, even after apparent recovery from a dysbiosis event, the microbiome may not fully return to its original state, potentially resulting in altered metabolic functions and long-term health consequences. Several pediatric autoimmune diseases have now been robustly linked to these patterns of microbial dysbiosis (Table 1).

In type 1 diabetes (T1D), longitudinal studies reveal that children who progress to clinical onset often manifest increased fungal and bacterial dysbiosis, accompanied by gut inflammation, well before hyperglycemia appears [96]. This dysbiosis is characterized by a reduction in SCFA-producing bacteria and an enrichment of proinflammatory genera, such as *Bacteroidetes* and *Prevotella*, which may disrupt oral tolerance and pancreatic islet autoimmunity.

Juvenile idiopathic arthritis (JIA), the most common pediatric rheumatic disease, exhibits similarly reduced microbial diversity and shifts in microbial composition, including increased abundance of Veillonella and Collinsella species [97]. These microbial changes are hypothesized to modulate systemic inflammation through altered gut permeability and translocation of microbial products, which activate the innate immune system and promote autoimmune joint inflammation.

Inflammatory bowel disease (IBD), including both Crohn’s disease and ulcerative colitis in children, shows profound dysbiosis typified by expansion of potentially pathogenic bacterial families, like *Veillonellaceae*, *Neisseriaceae*, and *Fusobacteriaceae*, alongside depletion of protective genera such as *Bacteroides* and *Faecalibacterium* [98,99]. This imbalance exacerbates aberrant immune responses to intestinal microbiota, sustaining chronic mucosal inflammation and autoimmunity.

Emerging data also link early microbiome perturbations to autoimmune thyroid diseases such as Graves’ disease in pediatric populations [100]. Adult studies show decreased beneficial bacteria, such as Bifidobacteria and *Lactobacilli*, in hyperthyroidism, whereas Hashimoto’s disease has a relative enrichment of *Akkermansia*, *Bifidobacterium*, and *Lachnospiraceae*. However, pediatric research during this critical period of microbial development remains limited.

Pediatric celiac disease, an autoimmune enteropathy triggered by gluten, is associated with distinct gut microbiota alterations. Children with celiac disease exhibit reduced populations of beneficial *Firmicutes* and increased proinflammatory *Proteobacteria*, which may influence intestinal barrier function and contribute to inappropriate immune activation against dietary gluten [94].

## 7. Interventions to Modulate the Early-Life Microbiome

Interventions targeting early-life gut dysbiosis have been developed to address disruptions associated with increased autoimmune disease risk (e.g., type 1 diabetes, celiac disease, inflammatory bowel disease) [91,92,93]. Observational data link cesarean delivery and preterm birth to modestly elevated T1D/IBD risks (odds ratio from 1.2 to 1.6) during neonatal immune programming [101,102]. These interventions aim to restore microbiome patterns disrupted by cesarean section or preterm birth—dysbiosis patterns previously linked to IBD through depletion of short-chain fatty acid (SCFA)-producing bacteria and to T1D/celiac tolerance via regulatory T cell induction mediated by microbial metabolites such as indole-3-lactic acid (ILA) produced by *Bifidobacterium* species [71,72,73]. However, no study to date reports autoimmune endpoints; microbiome normalization remains the primary outcome measure (see Table 2).

Dietary interventions, which modify the gut microbiota composition, have been investigated as non-invasive intervention strategies. For instance, in pediatric Crohn’s disease, exclusive enteral nutrition (EEN), which replaces normal dietary components with liquid nutrients, has been shown to improve disease activity and lead to remission but was also to associate with reduced α-diversity, depletion of *Faecalibacterium prausnitzii* and *Roseburia* (SCFA-producers), and enrichment of *Enterobacteriaceae* in approximately 50% of taxa compared to healthy controls [114]. Another example is the MELODY trial, which investigates the effects of an IBD anti-inflammatory diet, designed to favor SCFA-producing bacteria, versus control (no dietary intervention) in the third pregnancy trimester of mothers with Crohn’s disease. Primary and secondary outcomes include the maternal diet-induced changes in microbiome and CD activity and 1-year postpartum relapse, as well as the longitudinal changes in the infant’s gut microbiome diversity and relative abundance of *Proteobacteria* and *Bifidobacterium* [115].

Besides dietary interventions, administration of probiotics (live bacteria), prebiotics (fiber intake to foster the growth and function of beneficial gut microorganisms), or synbiotics (combination of prebiotics and probiotics) has been employed to target the microbiome functions.

Administration of probiotics, such as *Lactobacillus* and *Bifidobacterium* strains, increases beneficial taxa and reduces pathogens in preterm/CS infants. Numerous clinical trials show that supplementation with these probiotics leads to increases in beneficial gut bacteria, improvement in gut barrier function, decreases in pathogenic bacteria, and positive effects on immune development in infants [103,104,105,106]. Similarly, interventions with synbiotics, comprising *Bifidobacterium breve* M-16V in combination with the prebiotics short-chain galacto-oligosaccharides (scGOS) and long-chain fructo-oligosaccharides (lcFOS), have been shown to modulate the infant microbiome composition and specifically to restore CS-induced dysbiosis toward vaginal/breastfed patterns, and to benefit the microbiome of particularly formula-fed infants [107,108,109,110].

Another way to modulate the gut microbiome is via bacteriophages. Bacteriophages, or phages, are viruses that specifically target bacteria, often with a narrow host range that limits their impact on non-target species in the microbiome. This specificity has led to the development of phage therapies for various gastrointestinal conditions. There are currently no published clinical studies that directly use bacteriophages to modulate or replace the early-life microbiome in infants. However, in vitro phage cocktails have shown promise in treating *Clostridioides difficile* infections by eliminating the pathogen while preserving beneficial commensal bacteria such as bifidobacteria, enterococci, enterobacteria, and *lactobacilli* in vitro [116]. Similarly, in mice, bacteriophage therapy has been investigated to target adherent invasive *Escherichia coli* (AIEC) and *Klebsiella pneumoniae* and shown to reduce inflammation in mouse models of colitis [117,118,119]. However, due to the complex interactions within the gut microbiome, phage therapy can have unintended consequences. Studies have shown that targeting specific bacteria like *E. coli* and *Clostridium sporogenes* can indirectly affect non-target species and alter the gut metabolome [120]. The immunological impact of phages is another consideration. Phage DNA has been shown to stimulate IFN-γ production through a TLR9-dependent pathway, potentially increasing inflammation [120]. This underscores the importance of additional studies to clarify how phage therapy influences both the gut microbiome and the host, directly and indirectly. To date, no clinical studies have directly applied phage-based interventions to modulate the early-life microbiome in human infants.

Restoration strategies, such as vaginal microbial transfer (VMT) and maternal fecal microbiota transplantation (FMT), have been explored to address differences in microbial exposures between cesarean and vaginally delivered infants. Randomized controlled trials indicate that while both VMT and maternal FMT can partially normalize the gut microbiota of C-section-born infants, their effectiveness varies. VMT, which involves swabbing the infant with maternal vaginal fluids or oral administration, can introduce some maternal microbes—particularly increasing the abundance of genera such as *Bacteroides* and *Lactobacillus*—and may partially align the gut and skin microbiota of C-section-born infants with those of vaginally delivered infants [111,112]. However, the restoration is incomplete: VMT does not fully replicate the gut microbiota profile of vaginally delivered infants, and its effects on allergy risk, growth, or long-term health outcomes remain uncertain. The minimal effect is probably due to vaginal microbes being specialized for the vaginal environment and unable to persist long-term in the infant’s gut [111].

Recent evidence suggests that maternal FMT—administered orally to newborns shortly after birth—can more comprehensively normalize the gut microbiota of C-section-born infants, with restored abundance of key bacterial taxa, sustained effects for at least three months, and no serious adverse events [113].

The application of FMT is a rapidly evolving field, with growing interest in the development and clinical testing of customized bacterial consortia and next-generation microbial therapeutics as alternatives to traditional donor stool transplants. Several proof-of-concept and comparative studies indicate that, while maternal FMT appears effective and safe in term infants, broader application in pediatric populations remains limited by concerns about safety, transmission risks, and appropriate donor selection—factors especially relevant for immunocompromised or preterm children [113].

## 8. Discussion

This review highlights that early-life dysbiosis is a common feature across pediatric autoimmune diseases, including type 1 diabetes, inflammatory bowel disease, juvenile idiopathic arthritis, and celiac disease. Despite differences in disease mechanisms, these conditions consistently show overlapping microbial alterations: reduced diversity, depletion of beneficial taxa such as *Bifidobacterium* and SCFA producers (*Faecalibacterium*, *Roseburia*), and enrichment of pro-inflammatory or pathobiont-like taxa (*Enterobacteriaceae*, *Ruminococcus gnavus*) [91,92,93,94,95]. These shared dysbiosis patterns converge on a set of common immune disruptions—impaired regulatory T cell induction, reduced SCFA signaling, increased gut permeability, and subclinical gut inflammation—suggesting that interventions targeting these core functions may have broad preventive potential [68,70,71,72,73]. Interventions restoring these dysbiosis patterns (Table 2) represent promising preventive approaches, though long-term autoimmune outcomes remain untested.

Clinical and preclinical data indicate that interventions mimicking “natural” colonization, such as maternal fecal microbiota transplantation or specific synbiotics (e.g., scGOS/lcFOS in combination with *B. breve* M-16V, *B. infantis* EVC001), are more effective at normalizing microbiota composition and reducing inflammatory markers than generic probiotics or vaginal microbial transfer [103,104,105,106,107,108,109,110,111,112,113]. The timing and context of interventions are also crucial, with the greatest impact observed when applied during critical developmental windows and tailored to individual risk factors.

In addition to the limitations of 16S rRNA sequencing, which restricts resolution to the genus level and fail to capture functional data, virome, mycobiome, or metabolite contributions, several other factors complicate the study of early-life microbiome acquisition and its relationship to autoimmune disease risk. Indeed, the mother’s health status, ethnicity, geographical location, and dietary habits profoundly shape her microbiome and, consequently, the infant’s initial microbial colonization. For example, maternal obesity and gestational diabetes have been linked to newborn gut dysbiosis with reduced beneficial taxa and increased opportunistic bacteria [121], while maternal inflammatory bowel disease is associated with reduced *Bifidobacterium* and enrichment of pro-inflammatory taxa in the offspring [122]. Similarly, geographical and ethnic differences result in distinct infant microbial profiles, even under broadly similar environmental conditions [9,123]

The role of vertical transmission in the development of autoimmune diseases later in life remains an area of active investigation. While some studies suggest that maternal dysbiosis can transmit pathobionts and alter immune programming [122,124], the causal relationship between specific maternal microbiome features and infant autoimmune outcomes is not yet fully understood. Future research should aim to disentangle the effects of maternal health, ethnicity, geography, and diet on vertical transmission and autoimmune risk, using multi-omics approaches (shotgun whole-genome sequencing, transcriptomics, metabolomics) in longitudinal cohort studies. By considering these additional variables, researchers can gain a more nuanced understanding of the factors that influence early-life microbiome acquisition and develop more targeted and effective preventive strategies for autoimmune diseases.

## 9. Conclusions

The early-life gut microbiome plays a fundamental role in shaping immune system development and establishing tolerance. Disruptions in microbial colonization—due to cesarean delivery, antibiotic use, and formula feeding—lead to dysbiosis characterized by reduced diversity, loss of bacterial taxa enriched in healthy controls (e.g., *Bifidobacterium*, *Bacteroides*, and *Enterococcus faecalis*), and reduced abundance of short-chain fatty acid (SCFA)–synthesizing taxa, with consequent alterations in immunomodulatory metabolites such as SCFAs. While fecal SCFA levels reflect colonic production and may vary with intestinal transit time and absorption, dysbiosis consistently reduces the abundance of SCFA-synthesizing taxa, thereby altering host immunomodulation (e.g., Treg expansion). These alterations are linked to an increased risk of pediatric autoimmune diseases, including type 1 diabetes, inflammatory bowel disease, celiac disease, and juvenile idiopathic arthritis.

Current interventions, such as probiotics, synbiotics, vaginal microbial transfer, and maternal fecal microbiota transplantation, can partially restore a disrupted microbiome. However, while these approaches improve microbial composition and function, they have not yet been shown to directly prevent autoimmune disease onset in humans. Although the diminished abundance of certain gut commensal bacteria, such as *Bifidobacterium*, *Bacteroides*, and *Enterococcus faecalis*, appears to be a common feature in pediatric autoimmune conditions, an extra challenge in restoring the microbial composition is driven by the fact that metabolic capacities may differ at species and strain levels, requiring a deep characterization of the baseline microbiome structure for patient-specific interventions.

The early-life microbiome–immune interface may represent a promising target for the prevention of autoimmune diseases in childhood and adult life. Nonetheless, to truly establish the importance of the early-life “window of opportunity” in clinical settings, future studies must move beyond changes in microbiome structure and focus on long-term, longitudinal assessment of autoimmune outcomes following early microbiome modulation. Only then may we develop evidence-based, microbiome-targeted strategies to reduce the burden of chronic immune-mediated diseases.

## Figures and Tables

**Table 1:** Summary of pediatric diseases associated with perturbations in gut microbiome composition and functions; arrow ↓ = decreased; arrow ↑ = increase.

Diseases	Microbiome Alterations	Proposed Mechanisms	Ref.
Inflammatory bowel disease (IBD)	↓ Bacteroidetes/Firmicutes ratio; ↑ Gammaproteobacteria (*Enterobacteriaceae*, Fusobacterium); ↓ SCFA producers (Faecalibacterium, Roseburia)	Loss of barrier-protective and anti-inflammatory bacteria; increased oxidative stress and inflammation; impaired SCFA-mediated immune regulation	Morgan et al., 2012 [91]
Type 1 diabetes (T1D)	↓ Diversity; ↓ butyrate-producers (*Faecalibacterium*, *Lachnospiraceae*); ↑ pathobionts (*R. gnavus*, *S. infantarius*)	Impaired SCFA production, increased gut permeability, loss of immune tolerance, β-cell autoimmunity	Mariño et al., 2017, Vatanen et al., 2018 [92,93]
Celiac disease	↓ beneficial taxa; ↑ proteolytic pathogens (*P. aeruginosa*)	Altered antigen processing, increased inflammation, impaired oral tolerance	Leonard et al., 2021 [94]
Juvenile Idiopathic Arthritis (JIA)	↓ microbial richness; altered community structure; pro-arthritogenic profile	Subclinical gut inflammation, immune activation, and promotion of joint inflammation	van Dijkhuizen et al., 2019 [95]

**Table 2:** Early-life microbiome interventions: characteristics, outcomes, and autoimmune relevance. *CS: cesarean section; GA: gestational age; and MDRO: multidrug-resistant organisms.* Arrow ↓ = decreased; arrow ↑ = increase; w = weeks; mo = months.

Intervention	Strain/Dosage/Duration	Study Population	Primary Objective	Microbiome Outcome	Immune/Health Outcome	Autoimmune Relevance
Probiotic Supplementation [103]	*B. bifidum* + *L. acidophilus*, 2 × daily, first feed to 34 w GA	Preterm infants (*n* = 234)	Reduce NEC/sepsis via bifidogenic dominance	↑ *Bifidobacterium*, ↑ acetate/lactate, ↓ pathobionts	Lower fecal pH	SCFA production → Treg induction (T1D/IBD risk)
Probiotic supplementation [104]	*B. infantis*, *B. lactis*, *L. acidophilus*, first 28 days	Preterm 28–32 w GA (*n* = 618)	Reduce MDRO colonization	No MDRO reduction; diverse/stable microbiome	None reported	Diversity → immune tolerance
Probiotic supplementation [105]	*B. breve* PB04 + *L. rhamnosus* KL53A, 2 × 10^6^ *CFU/day, day 1–6*	CS-born term (*n* = 150)	Increase LAB, reduce pathogens	↑ *Lactobacillus*, *Bifidobacterium* (vaginal-like)	None reported	Early Bifidobacterium → SCFA/Treg axis
Probiotic supplementation [106]	*B. infantis* EVC001, 1.8 × 10^10^ CFU/day, 21 days	Breastfed term (*n* = 120)	Reduce enteric inflammation (0–6 w critical phase *)	↑ ILA 10×, stable colonization	↓ Pro-inflammatory cytokines (day 40/60)	ILA → Treg induction [71,72,73]
Synbiotic supplementation [107]	scGOS 0.02 g/g + *L. fermentum* CECT5716, 10^6^ CFU/g, 5 mo	Healthy term (*n* = 540)	Promote Bif/Lacto growth	↑ *Bifidobacterium*, *Lactobacillaceae*; ↓ *R. gnavus*	None reported	↓ Pathobionts → barrier function
Synbiotic supplementation [108]	scGOS/lcFOS 0.8 g/100 mL + *B. breve* M-16V 10^4^–10^6^ CFU/mL, 6 w	Formula-fed (*n* = 290)	↑ Bifidobacteria, ↓ pathogens	↑ *Bifidobacterium*; ↓ *C. difficile*	None reported	Bifidogenic dominance → stable/balanced microbiome
Synbiotic supplementation [109]	scGOS/lcFOS + *B. breve* M-16V, 17 w	Formula-fed, incl. CS (*n* = 224)	Restore CS dysbiosis	↑ *Parabacteroides* (17 w), *Bacteroides* (12 mo)	None reported	Bacteroides → weaning tolerance
Synbiotic supplementation [110]	scGOS/lcFOS + *B. breve* M-16V, birth-16 w	CS-born (*n* = 153)	Replicate vaginal/breastfed profile	↑ *Bifidobacterium*, ↑ acetate; ↓ *Enterobacteriaceae*	↓ Fecal pH	Stable/balanced microbiome restoration → tolerance
VMT [111]	Maternal vaginal gauze swab, immediate post-birth	7 vaginally delivered babies, 11 by CS (4 received VMT)	Partial vaginal microbiota transfer	Partial vaginal-like profile (skin still dominant)	None reported	Limited efficacy
VMT [112]	Maternal vaginal swab, immediate post-birth	177 babies, 98 born vaginally and 79 by CS, of whom 30 were swabbed	Restore vaginal trajectories	Trajectories closer to vaginal birth	None reported	Transient effect
FMT [113]	1.0 mL maternal feces + milk, first feed	CS-born term (*n* = 7)	Restore vaginal/breastfed-like profile	↑ *Bifidobacterium/Bacteroides*; ↓ opportunists (3 mo)	Safe, no AEs	Most effective for immune programming

* Critical phase: first 6 weeks (neonatal immune programming window). * No studies report direct autoimmune endpoints; all studies focus on dysbiosis patterns linked to immune tolerance (SCFA, Treg, intestinal barrier function) associated with ↓ T1D/IBD risk [91,92,93] *.

## Data Availability

No new data were created or analyzed in this study. Data sharing is not applicable to this article.

## Abbreviations

The following abbreviations are used in this manuscript:

APC	Antigen-Presenting Cells
CS	Cesarean Section
EEN	Exclusive Enteral Nutrition
FMT	Fecal Microbiota Transplantation
GALT	Gut-Associated Lymphoid Tissues
GA	Gestational Age
HMO(s)	Human Milk Oligosaccharides
IBD	Inflammatory Bowel Disease
IFNγ	Interferon gamma
IgA	Immunoglobulin A
IL (e.g., IL-10, IL-22, IL-23, IL-25)	Interleukin (various types)
ILC	Innate Lymphoid Cells
iNKT	Invariant Natural Killer T cells
JIA	Juvenile Idiopathic Arthritis
MHC-II	Major Histocompatibility Complex class II
MLN	Mesenteric Lymph Nodes
MAIT	Mucosal Associated Invariant T cells
NEC	Necrotizing Enterocolitis
NICU	Neonatal Intensive Care Unit
OTUs	Operational Taxonomic Units
pDC	Plasmacytoid Dendritic Cell
PP	Peyer’s Patches
PRR	Pattern-Recognition Receptors
REG3g	Regenerating islet-derived protein 3 gamma
RORγt	Retinoic-acid-receptor-related orphan receptor gamma t
SCFA(s)	Short-Chain Fatty Acid(s)
T1D	Type 1 Diabetes
Treg	Regulatory T cells
TEDDY	The Environmental Determinants of Diabetes in the Young (study)
VMT	Vaginal Microbial Transfer
